# Assessing the Impact of Metacognitive Therapy on OCD Severity and Beliefs: A Three‐Wave Multisite RCT With Panel Mediation Analysis

**DOI:** 10.1155/da/5060244

**Published:** 2026-10-15

**Authors:** Saeed Anwar, Romana Younas, Wejdan Deebani, Jiang Yingjie

**Affiliations:** ^1^ School of Psychology Northeast Normal University Changchun 130024 China nenu.edu.cn; ^2^ Jilin Provincial Higher Education Key Laboratory of Cognitive and Brain Science Changchun China; ^3^ State Key Laboratory of Cognitive Science and Mental Health Institute of Psychology Chinese Academy of Sciences Beijing 100101 China cas.cn; ^4^ Department of Psychology University of Chinese Academy of Sciences Beijing China ucas.ac.cn; ^5^ Department of Mathematics Faculty of Science and Arts King Abdul Aziz University Rabigh Saudi Arabia kau.edu.sa

**Keywords:** metacognitive beliefs, metacognitive therapy, obsessive–compulsive disorder, randomized controlled trial, structural equation modeling

## Abstract

Obsessive–compulsive disorder (OCD) is a chronic and disabling condition, and although exposure and response prevention (ERP) is the first‐line psychological treatment, many patients decline it, drop out, or retain residual symptoms. According to Wells’ metacognitive model, OCD is maintained less by intrusive thoughts themselves than by metacognitive beliefs about the significance, danger, and control of thoughts, which metacognitive therapy (MCT) targets directly. However, individual MCT has been evaluated almost exclusively in high‐income Western settings, and whether metacognitive belief change temporally precedes symptom improvement remains untested. This multisite, assessor‐blinded randomized controlled trial (RCT) addressed both gaps in Pakistani outpatient services. Seventy‐six adults with OCD were randomized to 12 weekly sessions of individual MCT (*n* = 38) or documented treatment as usual (TAU; *n* = 38) and assessed at baseline, post‐treatment, and 6‐month follow‐up; outcomes were analyzed with intention‐to‐treat (ITT) mixed‐effects models and an exploratory three‐wave panel mediation model. MCT produced greater reductions in clinician‐rated Yale‐Brown Obsessive‐Compulsive Scale severity than TAU, *F*(1.81, 134) = 11.30, *p* < 0.001, with mean differences of −4.00 at post‐treatment and −6.92 at follow‐up (both *p* < 0.001) and more frequent treatment response. MCT also reduced Metacognitions Questionnaire‐30 belief domains, with gains maintained at follow‐up. However, the prespecified lagged indirect effect through post‐treatment beliefs was not supported (*β* = −0.01). Findings extend the evidence for MCT to a non‐Western clinical context and suggest that broad metacognitive belief domains behave as markers of clinical state rather than proximal mechanisms, underscoring the need for temporally fine‐grained mechanism research.

## 1. Introduction

Obsessive–compulsive disorder (OCD) is characterized by intrusive, unwanted thoughts, images, or urges (obsessions) and repetitive behaviors or mental acts (compulsions) performed to reduce distress or prevent feared outcomes (American Psychiatric Association [1]). OCD typically begins in adolescence or early adulthood and may follow a persistent course with substantial functional impairment, comorbidity, and reduced quality of life when symptoms are moderate to severe [2]. Although effective treatments exist, epidemiological and service‐use data indicate that many people with OCD do not receive adequate evidence‐based care, with particularly low treatment contact reported in many low‐ and middle‐income country settings [3, 4]. Evaluating specialist psychotherapies in settings that are underrepresented in the evidence base can show whether their clinical effects and theoretical predictions generalize across cultural and service contexts. Such trials do not, by themselves, establish scalability, affordability, or population‐level access.

Cognitive‐behavioral therapy with exposure and response prevention (ERP) remains the first‐line psychological treatment for OCD and is supported by systematic reviews and meta‐analyses of randomized controlled trials (RCTs) [5–7]. Nevertheless, ERP is not universally available, acceptable, or sufficient for all patients. It requires specialist competence, systematic exposure practice, response prevention, supervision, and sustained patient engagement, and adherence difficulties are common in routine care [8]. These considerations provide a clinical rationale for evaluating theoretically distinct interventions that may function as alternatives or complements to ERP.

Metacognitive therapy (MCT) for OCD, as evaluated in the present trial, is a therapist‐led, formulation‐based psychotherapy delivered in one‐to‐one sessions. It is derived from Wells’ metacognitive model and the Self‐Regulatory Executive Function (S‐REF) framework, which propose that emotional disorder is maintained not primarily by the content of intrusive thoughts but by a perseverative style of responding to internal experiences [9–11]. Central to this account is the Cognitive‐Attentional Syndrome (CAS), a pattern of threat monitoring, repetitive negative thinking, thought‐control attempts, reassurance seeking, checking, washing, and other maladaptive coping strategies that are guided by metacognitive beliefs about the meaning, controllability, and danger of thoughts [9, 11]. In MCT, the therapist develops an idiosyncratic formulation of these maintaining processes and uses this formulation to select metacognitive techniques and behavioral experiments for the individual patient.

Applied to OCD, the metacognitive model assumes that intrusive thoughts are common and not inherently pathological; obsessional distress is maintained when intrusions are interpreted through beliefs that thoughts are dangerous, meaningful, uncontrollable, or must be neutralized [10, 12, 13]. Compulsions, reassurance‐seeking, mental checking, washing, and thought suppression are therefore conceptualized as CAS‐linked coping strategies that may briefly reduce distress but maintain the perceived importance of intrusions and prevent flexible disengagement over time [10, 12]. The core therapeutic targets in Wells‐based MCT are therefore metacognitive beliefs about intrusive thoughts, beliefs about the necessity or usefulness of rituals and neutralizing rigid stop signals, and the patient’s habitual response style to intrusions.

The efficacy evidence for the Wells‐based MCT provides a direct rationale for testing this intervention in an RCT. In the original case series, Fisher and Wells [12] reported clinically significant improvement across four consecutively referred adults with OCD, with gains largely maintained at a 6‐month follow‐up. Subsequent randomized work extended this preliminary evidence. Glombiewski et al. [14] randomized 37 outpatients to 14 weekly sessions of MCT or ERP and found large reductions in OCD symptoms in both conditions, no statistically significant between‐treatment difference, and less therapist face‐to‐face time in the MCT condition. Melchior et al. [15] then randomized 90 outpatients to MCT or ERP and found significant reductions in obsessive–compulsive symptoms, comorbid psychological symptoms, and metacognitive beliefs in both groups, with moderate‐to‐large within‐group effects, low dropout, high rates of clinically significant change, and maintenance of gains at 6‐month follow‐up. Most recently, Exner et al. [16] randomized 74 outpatients to 12 weekly sessions of manualized MCT or ERP in a non‐inferiority trial and reported low dropout rates, comparable Y‐BOCS improvements, and non‐inferiority of MCT to ERP at post‐treatment, although non‐inferiority was not confirmed at the 6‐month follow‐up. Taken together, the available evidence suggests that MCT is a plausible specialist psychotherapy for OCD while also indicating that further trials are needed in settings outside the high‐income Western contexts from which most evidence has emerged.

Other OCD interventions also use the term metacognitive, but they represent different treatment formats. My metacognitive training for OCD (myMCT; [17]) was developed as a self‐help manual for independent use and is organized around psychoeducational exercises that patients can apply without continuous therapist‐led case formulation. Metacognitive training for OCD (MCT–OCD), developed by Miegel, Jelinek, Moritz, and colleagues, is a highly structured, group‐based metacognitive training program delivered using standardized, slide‐supported materials and modular exercises [18–20]. Its modules target OCD‐relevant dysfunctional beliefs, cognitive biases, metacognitions, and coping strategies, including perfectionism, intolerance of uncertainty, fusion beliefs, control of thoughts, overestimation of threat, inflated responsibility, biased cognitive networks, and biased attention.

The distinction among these approaches is therefore conceptual and procedural, not merely a difference between individual and group delivery. Wells‐based MCT is an individualized psychotherapy guided by a patient‐specific metacognitive case formulation and an active therapist role; myMCT is a self‐help manual intended for independent use; and MCT‐OCD is a standardized group training format with modular psychoeducational content. These formats differ in delivery mode, degree of standardization, therapist involvement, and content focus. The present trial evaluated the individual therapist‐delivered format rather than the myMCT or MCT–OCD for two reasons. First, the strongest randomized efficacy evidence for a Wells‐based metacognitive approach to OCD comes from individually delivered, formulation‐based MCT tested directly against ERP [14–16], whereas myMCT and MCT–OCD were developed as a self‐help manual and a standardized group training program, respectively, and therefore address a different delivery question [17–20]. Second, and central to the aims of this study, testing whether metacognitive belief change temporally precedes symptom improvement requires an individualized case formulation in which the therapist can identify and modify each patient’s specific metacognitive beliefs and ritual‐maintaining processes, so that belief change can be tracked as a putative mechanism rather than delivered as fixed, pre‐scripted psychoeducational content. The individual format was therefore the format most suited to a mechanism‐focused, outpatient effectiveness trial.

A central unresolved issue is whether changes in metacognitive beliefs actually function as mechanisms of later symptom improvement. MCT is designed to modify maladaptive metacognitive beliefs, and prior research indicates that metacognitive beliefs can change during OCD treatment and are associated with symptom outcomes [21, 22]. However, improvement in both beliefs and symptoms does not establish that belief change temporally precedes symptom change. Symptoms may improve first and subsequently alter belief endorsement, or both processes may change in parallel because of shared therapeutic learning.

For this reason, mechanism testing requires designs that separate treatment effects, changes in the mediator, and changes in the later outcome. Psychotherapy mechanism research emphasizes that mediation claims are strongest when the proposed mediator is measured before the later outcome, prior outcome levels are controlled, and competing temporal directions are tested [23–25]. These requirements are especially important in OCD, where prior studies have shown that cognitive and behavioral process variables may change concurrently with symptoms or may be predicted by earlier symptom improvement rather than clearly preceding it [26–28]. A three‐wave panel mediation model therefore provides a more stringent test of whether post‐treatment metacognitive beliefs account for subsequent OCD severity. Two gaps therefore remain in the individual‐MCT literature: this treatment has been evaluated almost entirely in high‐income Western settings, leaving its effectiveness in other cultural and service contexts largely untested, and its proposed metacognitive mechanism has rarely been examined with a temporally ordered design that can establish whether belief change precedes symptom change. The present trial was designed to address both gaps.

In the present RCT, adults with OCD were randomized to 12 weekly sessions of manual‐guided MCT or documented treatment as usual (TAU), with assessments at baseline, post‐treatment, and 6‐month follow‐up. The study had three aims. First, we evaluated whether MCT produced greater reductions in clinician‐rated OCD severity than TAU using intention‐to‐treat (ITT) mixed‐effects models. Second, we tested whether MCT produced greater reductions in dysfunctional metacognitive beliefs across the five Metacognitions Questionnaire‐30 domains. Third, we examined whether post‐treatment metacognitive beliefs mediated the effect of treatment on follow‐up OCD severity in a temporally ordered three‐wave panel model that controlled for baseline beliefs and post‐treatment OCD severity.

Based on the metacognitive model and previous outcome evidence, we hypothesized that MCT would reduce OCD severity more than TAU and would produce greater reductions in dysfunctional metacognitive beliefs [10, 12, 15, 16]. We further hypothesized that lower post‐treatment metacognitive belief endorsement would mediate the effect of treatment on lower OCD severity at follow‐up, while recognizing that the panel model provided a conservative test because it adjusted for earlier symptom severity and allowed reciprocal symptom‐belief pathways to be examined.

## 2. Methods

### 2.1. Study Design

This multisite, assessor‐blinded, parallel‐group RCT evaluated individualized, manual‐guided MCT versus documented TAU for adults with OCD in Pakistan. The active intervention was a formulation‐based MCT protocol grounded in Wells’ metacognitive model and delivered in 12 weekly individual sessions. TAU was documented using a usual care guide and session checklists. Participants were allocated 1:1 to MCT or TAU and were assessed at baseline (T0), immediately post‐treatment after the 12‐session intervention phase (T1), and at 6‐month follow‐up (T2). The primary objective was to test whether MCT produced greater reductions in clinician‐rated OCD severity (Yale–Brown Obsessive‐Compulsive Scale [Y‐BOCS]) over time compared with TAU. Secondary objectives were to evaluate treatment effects on dysfunctional metacognitive beliefs (Metacognitions Questionnaire‐30 [MCQ‐30]) and to test a hypothesized mechanism of change using a temporally ordered three‐wave panel mediation model in which post‐treatment metacognitive beliefs (T1) were specified as parallel mediators of the treatment effect on follow‐up OCD severity (T2).

### 2.2. Participants and Recruitment

Participants were recruited from two outpatient clinical settings in Islamabad, Pakistan. Recruitment was conducted in collaboration with clinical staff at both sites. Prior to enrollment, the research team met with administrators and clinicians to review the study aims, eligibility criteria, assessor blinding procedures, confidentiality safeguards, and standardized data collection procedures. Clinicians introduced the study to potentially eligible patients during routine appointments and referred interested individuals to trained research assistants. Patients were informed that participation was voluntary and that declining participation would not affect their clinical care. After providing written informed consent, participants completed baseline questionnaires, including the Symptom Checklist‐90 (SCL‐90) to screen for broader comorbid symptom burden, and underwent a structured clinical interview to confirm the DSM‐5 diagnosis of OCD and eligibility. In total, 120 individuals were screened, and 76 eligible participants were randomized (MCT: *n* = 38 and TAU: *n* = 38).

### 2.3. Inclusion and Exclusion Criteria

Inclusion criteria were: (a) age ≥18 years; (b) a primary diagnosis of OCD based on DSM‐5 criteria, confirmed by a licensed psychiatrist through clinical evaluation and structured diagnostic interview; and (c) baseline clinician‐rated OCD severity in the moderate range or higher (Y‐BOCS total score ≥16). Participants with comorbid psychiatric disorders and/or psychotropic medication use were eligible if clinically stable, defined as no medication dosage change for at least 6 weeks prior to baseline. Exclusion criteria were: current psychotic disorder or bipolar disorder; a major depressive episode requiring immediate treatment; substance use disorder requiring specialist treatment; intellectual disability or significant cognitive impairment; concurrent psychotherapy for OCD or another anxiety disorder; medication changes within 6 weeks before baseline; or receipt of evidence‐based OCD treatment within the 3 months prior to baseline. Eligibility was determined by applying these criteria during the structured screening assessment, and the resulting participant flow is summarized in Figure 1. Of the 120 individuals assessed for eligibility, 44 were not randomized: 4 were excluded because they met the exclusion criterion of a current psychotic disorder, 16 were excluded because they had a change in psychotropic medication within 6 weeks before baseline, and 24 declined to participate and were therefore not enrolled. The remaining 76 participants met all inclusion criteria, met none of the exclusion criteria, and provided written informed consent; these participants were randomized 1:1 to MCT (*n* = 38) or TAU (*n* = 38).

**Figure 1:**
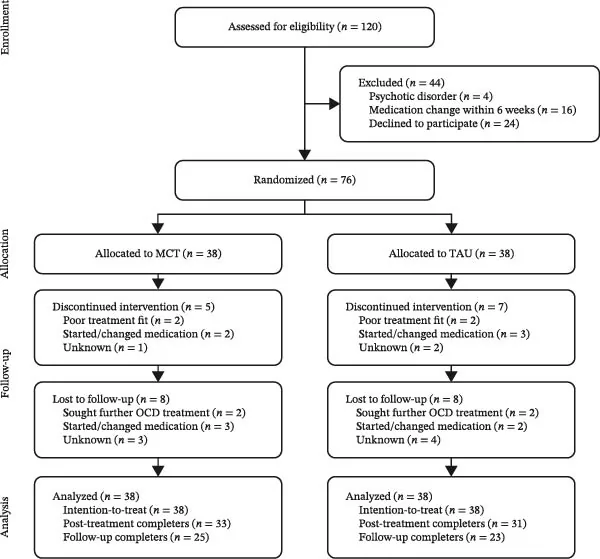
Flowchart of the study. MCT, metacognitive therapy; TAU, treatment as usual.

### 2.4. Measures

#### 2.4.1. Y‐BOCS

The primary outcome was clinician‐rated OCD symptom severity measured with the Y‐BOCS [29], a semi‐structured interview consisting of 10 items (5 obsession severity and 5 compulsion severity). Items were rated from 0 (none) to 4 (extreme), yielding a total score from 0 to 40, with higher scores indicating greater severity. Trained outcome assessors administered the Y‐BOCS at baseline (T0), post‐treatment (T1), and follow‐up (T2). In the present trial, baseline internal consistency was acceptable (Cronbach’s *α* = 0.79).

#### 2.4.2. MCQ‐30

Dysfunctional metacognitive beliefs were assessed using the MCQ‐30 [30]. The MCQ‐30 yields five domain scores: (a) cognitive confidence, (b) positive beliefs about worry, (c) cognitive self‐consciousness, (d) negative beliefs about uncontrollability and danger, and (e) beliefs about the need to control thoughts. Items were rated on a 4‐point Likert scale, with higher scores reflecting stronger endorsement of dysfunctional metacognitive beliefs. Participants completed the MCQ‐30 at T0, T1, and T2.

#### 2.4.3. SCL‐90

To screen for broader comorbid psychological symptom burden and index general psychopathology beyond OCD, participants completed the SCL‐90 [31] at pretreatment (T0). The SCL‐90 is a 90‐item self‐report inventory rated on a 5‐point Likert scale from 1 (not at all) to 5 (very much), yielding a total score range of 90–450, with higher scores indicating greater general symptom severity. Although the SCL‐90 is not a diagnostic instrument, it provides a broad assessment of common symptom domains and therefore supports screening for comorbid complaints in clinical samples. In the present trial, internal consistency at pretreatment (T0) was excellent (Cronbach’s *α* = 0.93).

### 2.5. Procedure and Ethical Considerations

#### 2.5.1. Procedure and Assessment Schedule

After providing written informed consent, participants completed baseline self‐report measures and a diagnostic assessment to confirm their eligibility. Following randomization, participants received either 12 weekly therapist‐delivered MCT sessions or TAU. Post‐treatment assessments (T1) were conducted immediately after the 12‐week treatment phase, and follow‐up assessments (T2) were conducted 6 months after treatment completion. Clinician‐rated outcomes were administered by trained assessors who were blinded to treatment allocation, and self‐report measures were completed independently by participants. Potential harms and clinical deterioration were monitored through participant report, therapist notes, outcome‐assessment contacts, and weekly supervision; monitored events included symptom worsening requiring urgent intervention, psychiatric hospitalization, suicide attempt, treatment‐related clinical crisis, medication emergency, and discontinuation due to an adverse event.

#### 2.5.2. Randomization, Allocation Concealment, and Blinding

Randomization was performed at a 1:1 ratio using block randomization with a computer‐generated allocation sequence prepared by an independent researcher. Allocation was concealed using sequentially numbered opaque envelopes that were opened only after the completion of baseline assessments. Outcome assessors were blinded to the treatment condition, and participants were instructed not to disclose their assignment during assessments.

#### 2.5.3. Interventions and Treatment Fidelity

Four supervised clinical psychologists delivered the study interventions. Two psychologists delivered the MCT condition and two delivered the TAU condition, and no therapist worked across both conditions. Before recruitment, all therapists were trained in the study procedures and oriented to the condition‐specific session guides and fidelity checklists. Weekly on‐site supervision was led by the principal investigator to review case formulation, adherence to the assigned condition, and management of clinical issues.

Treatment fidelity was monitored using condition‐specific session checklists and therapy notes completed for all delivered sessions and reviewed during weekly on‐site supervision by the principal investigator. These records were used to verify the delivery of the assigned condition and to identify protocol deviations or cross‐condition contamination. No major protocol deviations or cross‐condition contamination were identified. Sessions were not audio‐ or video‐recorded, and independent external adherence or competence ratings and numerical competence scores were not obtained. Fidelity monitoring therefore relied on systematic session documentation and weekly supervisory review.

##### 2.5.3.1. MCT

Participants allocated to MCT received 12 weekly individual sessions of 45–60 min. The treatment was manual‐guided and based on Wells’ metacognitive model for OCD, as described in Fisher and Wells [12], Wells [10], and the OCD MCT protocol and trial report by Melchior et al. [15, 32]. Material was delivered directly by the psychologist in sessions through psychoeducation, collaborative case formulation, guided verbal methods, behavioral experiments, homework review, and relapse‐prevention planning. A typical MCT session began with a brief review of recent intrusive thoughts, rituals, or other neutralizing responses, and between‐session practice. The therapist then worked on the current metacognitive target identified in the formulation, using guided questioning and in‐session exercises or behavioral experiments when indicated, and ended the session by summarizing the learning points and agreeing on between‐session practice. The therapist’s role was active and individualized: identifying the patient’s CAS processes (e.g., threat monitoring, thought suppression, reassurance seeking, checking, washing, or covert neutralizing), eliciting the metacognitive beliefs and stop signals that maintained these responses, and selecting techniques to modify those maintaining processes.

The 12‐session protocol was organized into four formulation‐based modules consistent with Wells’ MCT model and the MCT protocol and trial literature [10, 12, 15, 32]. Module 1 (sessions 1–2) involved case formulation and socialization to the metacognitive model. The therapist normalized intrusive thoughts, elicited metacognitive beliefs about intrusions and rituals, identified maladaptive coping responses, and introduced detached mindfulness using psychoeducation, guided questioning, and thought suppression demonstrations. Module 2 (sessions 3–6) targeted beliefs about intrusive thoughts, including fusion and threat meanings attached to intrusions. Core procedures included verbal reattribution and metacognitive behavioral experiments. When clinically indicated, exposure and response commission and postponement, or exclusion of neutralizing responses, were used to test whether thoughts required control or ritualized responses. These procedures were not delivered as ERP because their therapeutic target was a change in metacognitive beliefs rather than fear habituation or conventional exposure‐based response prevention.

Module 3 (sessions 7–9) focused on beliefs about rituals and neutralizing, especially predictions that rituals are necessary, useful, or protective. The therapists used guided questioning, ritual‐modulation experiments, planned withholding of rituals, and reduction of covert neutralizing to test these predictions. These ritual‐focused procedures were implemented as metacognitive behavioral experiments rather than as formal ERP assignments. Module 4 (sessions 10–12) consolidated learning through a new response plan for intrusive experiences, revision of stop signals and checking criteria when relevant, relapse‐prevention planning, and a therapy blueprint summarizing the case formulation, disconfirmed metacognitive beliefs, central behavioral evidence, and an individualized plan for responding to future intrusions. Across modules, the therapist role was active and formulation‐guided rather than didactic alone, which distinguishes this protocol from slide‐supported group metacognitive training formats [10, 15, 18].

##### 2.5.3.2. TAU

TAU was operationalized as documented routine clinical care rather than manualized CBT, ERP, or metacognitive intervention. Participants allocated to TAU were offered 12 weekly individual sessions of 45–60 min, matching the planned frequency and duration of the MCT. TAU followed a brief investigator‐developed usual care guide and session checklist and comprised psychoeducation about OCD, supportive counseling, stress‐management guidance, and behavioral activation when clinically indicated. Formal ERP was not used in TAU: no TAU session included a planned exposure hierarchy, therapist‐directed exposure task, response‐prevention assignment, or metacognitive behavioral experiment. TAU also did not include individualized CBT or metacognitive case formulation, detached mindfulness, metacognitive belief‐modification procedures, exposure and response commission, ritual‐modulation experiments, modification of metacognitive stop signals, or a metacognitive relapse‐prevention blueprint. Any behavioral guidance was limited to general supportive clinical management and was not delivered according to an ERP or a MCT protocol.

#### 2.5.4. Ethical Approval, Trial Registration, and Confidentiality

The study protocol was approved by the Institutional Review Board of Northeast Normal University (IRB #2022035) and registered at ClinicalTrials.gov (NCT07052812). The registry entry lists the study as a completed, two‐arm randomized trial with planned enrollment of 76 participants, a January 2024 study start date, and change in the Y‐BOCS total score as the primary outcome. Public trial registration was completed after the trial period; therefore, the study should be interpreted as retrospectively registered. The primary clinical outcome and assessment schedule were specified in the IRB‐approved study protocol before data collection, whereas the three‐wave PLS‐SEM mediation model was specified before the final statistical analyses and is reported as a secondary mechanism analysis rather than as a prospectively registered primary analysis. The trial was conducted in accordance with CONSORT reporting guidelines, and the full study protocol is available from the corresponding author upon reasonable request. All participants provided written informed consent prior to participation and were informed that they could withdraw at any time without affecting their clinical care. To protect confidentiality, study data were identified using participant codes rather than direct identifiers, and access to study records was restricted to the research team.

### 2.6. Statistical Analysis

Analyses were planned to address three questions: (1) whether MCT produced greater reductions in clinician‐rated OCD severity over time compared with TAU; (2) whether MCT produced greater reductions in dysfunctional metacognitive beliefs than TAU; and (3) whether post‐treatment metacognitive beliefs temporally mediated the treatment effect on follow‐up OCD severity.

For aims (1) and (2), primary analyses followed the ITT principle and included all randomized participants. Treatment effects were evaluated in GraphPad Prism 10.6.1 using restricted maximum likelihood (REML) mixed‐effects models for repeated measures. Separate models were specified for the Y‐BOCS total scores and each of the five MCQ‐30 domain scores. Each model included fixed effects for treatment condition (MCT vs. TAU), time (categorical: baseline, post‐treatment, and follow‐up), and the condition × time interaction, with participant specified as the repeated/random subject factor. Missing repeated‐measures data were handled under a missing‐at‐random framework. For analyses requiring a complete rectangular data matrix, including categorical response/remission summaries and the PLS‐SEM panel mediation model, missing values were handled in R using multiple imputation by fully conditional specification. The imputation model included randomized condition, recruitment site, available baseline demographic and clinical characteristics, and all available wave‐specific Y‐BOCS and MCQ‐30 domain scores. Sphericity was not assumed. Therefore, Prism’s Geisser‐Greenhouse adjustment was used for the numerator and denominator degrees of freedom for the within‐person time effect and the condition × time interaction; the between‐person condition main effect was tested using the corresponding between‐subject degrees of freedom. The condition main effect was interpreted only as the average between‐group difference across all assessment occasions; the primary treatment hypothesis was evaluated using the condition × time interaction and the planned baseline, post‐treatment, and follow‐up simple‐effect contrasts. Planned contrasts were corrected for multiple comparisons using Tukey or Šídák procedures as appropriate and are reported with model‐estimated mean differences and 95% confidence intervals.

For clinical interpretation, Y‐BOCS response and remission were evaluated separately at post‐treatment and 6‐month follow‐up. Response was defined as a reduction of at least 35% from baseline Y‐BOCS severity, and remission was defined as an endpoint Y‐BOCS total score ≤12. These thresholds represent the Y‐BOCS components of the international consensus definitions for OCD treatment response and remission [33]. Because Clinical Global Impression‐Improvement (CGI‐I) and Clinical Global Impression‐Severity (CGI‐S) ratings were not collected, the outcomes are reported as Y‐BOCS‐based response and remission rather than the full consensus classifications. Rates were summarized as *n*/*N* (%) by treatment condition using the repeated‐measures ITT analysis dataset. Between‐group effects were reported as absolute risk differences with Newcombe‐Wilson 95% confidence intervals [34], risk ratios, and odds ratios. A 0.5 Haldane‐Anscombe continuity correction was applied to risk‐ratio and odds‐ratio estimates when one condition had no events. When neither condition had an event, ratio estimates were reported as not estimable.

For aim (3), mechanism hypotheses were examined using a longitudinal three‐wave panel mediation model to enforce temporal ordering. The hypothesized three‐wave panel mediation model is shown in Figure 2. In this model, treatment condition (T0) predicted post‐treatment metacognitive beliefs (T1), which were specified as parallel mediators of follow‐up OCD severity (T2) while controlling for baseline belief levels (T0) and post‐treatment OCD severity (T1). The model was estimated using partial least‐squares structural equation modeling (PLS‐SEM) in SmartPLS 4.1.1.4, with indirect effects evaluated via bias‐corrected bootstrap confidence intervals (5000 resamples). Because PLS‐SEM requires complete data, missing values in variables included in the panel model were imputed using multiple imputation by fully conditional specification under a missing‐at‐random assumption; treatment condition, baseline characteristics retained in the analysis dataset, and all wave‐specific Y‐BOCS and MCQ‐30 scores were included in the imputation model. The primary PLS‐SEM analysis therefore retained the full randomized sample (*N* = 76). A complete‐case model (participants with complete data at all waves; *n* = 48) was estimated as a sensitivity analysis. Given the number of parallel mediators and longitudinal control paths relative to the sample size, the mediation model was treated as exploratory and hypothesis‐generating rather than as a definitive causal test.

**Figure 2:**
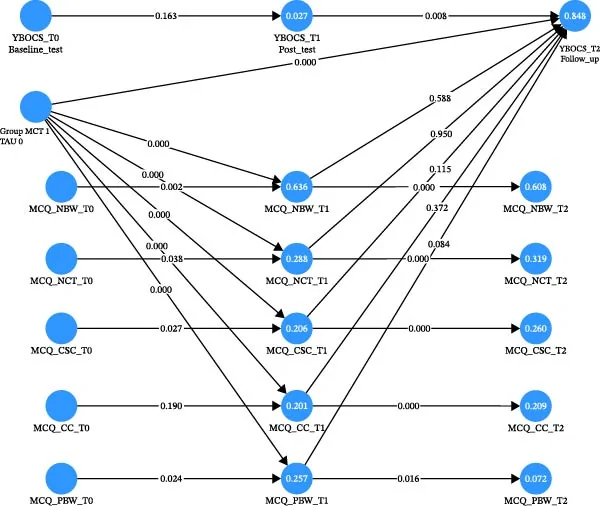
Three‐wave panel mediation model testing metacognitive beliefs as mechanisms of change in OCD severity. *Note:* T0 = baseline; T1 = post‐treatment; T2 = 6‐month follow‐up. Y‐BOCS = Yale–Brown Obsessive–Compulsive Scale. MCQ‐30 domains; NBW = negative beliefs about uncontrollability and danger; CC, cognitive confidence; CSC, cognitive self‐consciousness; NCT, need to control thoughts; PBW, positive beliefs about worry. Group indicates randomized condition (coded as shown in the figure: 1 = MCT, 0 = TAU). Values inside endogenous nodes represent *R* ^2^ (variance explained). Numbers printed on single‐headed arrows are *p*‐values for the corresponding structural paths; values below 0.001 are reported as *p* < 0.001.

As an additional sensitivity test of reverse temporal ordering, we estimated a model in which post‐treatment OCD severity (T1) predicted follow‐up metacognitive beliefs (T2), controlling for baseline and post‐treatment belief levels. Finally, an a priori power analysis indicated that a sample size of 76 would provide ~80% power to detect medium‐sized between‐group effects (Cohen’s *d* = 0.50) at *α* = 0.05 (two‐tailed) for the primary treatment effect aims. By contrast, the panel mediation analysis was statistically more demanding because it included five parallel mediators, autoregressive and cross‐lagged paths, a direct treatment path, and imputed follow‐up observations. Therefore, the PLS‐SEM mediation model was treated as an exploratory, hypothesis‐generating test of temporal mechanisms rather than a confirmatory test of causal mediation. To evaluate the model’s out‐of‐sample predictive validity, which is considered more diagnostic than approximate global fit indices for variance‐based PLS‐SEM [35], we conducted PLSpredict with a linear model (LM) benchmark and the cross‐validated predictive ability test (CVPAT) against an indicator‐average (IA) benchmark [36, 37].

## 3. Results

### 3.1. Baseline Characteristics and Attrition

A total of 120 individuals were screened, of whom 76 were randomized to receive either MCT (*n* = 38) or TAU (*n* = 38). Of the randomized participants, 41 (53.9%) were recruited from outpatient clinical site 1 and 35 (46.1%) from outpatient clinical site 2, with similar site representation across conditions. The flow of participants through screening, randomization, treatment, and follow‐up is presented in Figure 1. Twelve participants discontinued treatment during the active intervention phase (MCT, *n* = 5; TAU, *n* = 7), with no evidence of differential dropout by condition (Fisher’s exact *p* = 0.75). At the 6‐month follow‐up, 48 participants (63.2% of those randomized) completed the clinician‐rated Y‐BOCS assessment (MCT: *n* = 25; TAU: *n* = 23), and follow‐up completion did not significantly differ by condition (Fisher’s exact *p* = 0.81). Baseline demographic and clinical characteristics, including recruitment site, general psychopathology, recorded psychiatric comorbidity, medication status, OCD duration, family history, and baseline Y‐BOCS severity, are summarized by condition in Table 1. Baseline metacognitive belief scores are reported with the repeated‐measures outcome means in Table 2. All analyses followed the ITT approach, with missing data handled as described in Section 2.6.

**Table 1:** Baseline demographic and clinical characteristics of randomized participants.

Characteristic	Total sample (*N* = 76)	MCT (*n* = 38)	TAU (*n* = 38)
Outpatient clinical site 1, *n* (%)	41 (53.9)	21 (55.3)	20 (52.6)
Outpatient clinical site 2, *n* (%)	35 (46.1)	17 (44.7)	18 (47.4)
Age, years, mean (SD)	32.87 (7.87)	32.45 (7.63)	33.29 (8.14)
Male, *n* (%)	43 (56.6)	21 (55.3)	22 (57.9)
Female, *n* (%)	33 (43.4)	17 (44.7)	16 (42.1)
OCD duration, years, mean (SD)	6.96 (3.48)	6.82 (3.41)	7.10 (3.58)
Baseline Y‐BOCS total, mean (SD)	31.20 (3.75)	30.84 (4.14)	31.55 (3.33)
Baseline SCL‐90 total, mean (SD)	205.74 (44.18)	203.16 (43.55)	208.32 (45.10)
OCD family history, yes, *n* (%)	26 (34.2)	13 (34.2)	13 (34.2)
OCD family history, no, *n* (%)	50 (65.8)	25 (65.8)	25 (65.8)
Any comorbid psychiatric disorder, *n* (%)	29 (38.2)	14 (36.8)	15 (39.5)
No comorbid psychiatric disorder, *n* (%)	47 (61.8)	24 (63.2)	23 (60.5)
Depressive disorder, *n* (%)	13 (17.1)	6 (15.8)	7 (18.4)
Anxiety‐related disorder other than OCD, *n* (%)	10 (13.2)	5 (13.2)	5 (13.2)
Other recorded psychiatric disorder, *n* (%)	6 (7.9)	3 (7.9)	3 (7.9)
Current psychotropic medication use, *n* (%)	19 (25.0)	8 (21.1)	11 (28.9)
No current psychotropic medication use, *n* (%)	57 (75.0)	30 (78.9)	27 (71.1)
Stable medication dose ≥6 weeks before baseline, *n* (%)	19 (25.0)	8 (21.1)	11 (28.9)

*Note*: Recruitment‐site labels identify the two outpatient clinical settings in Islamabad. Recorded psychiatric comorbidity reflects diagnoses documented during the baseline clinical assessment. Medication status refers to psychotropic medication use recorded at baseline. Participants using psychotropic medication were eligible only if clinically stable, defined as no medication dosage change for at least 6 weeks before baseline. Medication use was not part of either assigned psychotherapy condition.

Abbreviations: MCT, metacognitive therapy; OCD, obsessive–compulsive disorder; SCL‐90, Symptom Checklist‐90; TAU, treatment as usual; Y‐BOCS, Yale‐Brown Obsessive‐Compulsive Scale.

**Table 2:** Compact mixed‐effects treatment estimates for OCD severity and MCQ‐30 domains.

Outcome and condition × time test	Time point	MCT *M* (SD)	TAU *M* (SD)	MCT–TAU difference (95% CI)	*p*	Hedges’ *g*
Y‐BOCS severity *F*(1.81, 134) = 11.30, *p* < 0.001	Baseline	30.8 (4.1)	31.6 (3.3)	−0.71 (−2.43, 1.01)	0.413	−0.19
	Post‐treatment	25.2 (3.9)	29.2 (3.1)	−4.00 (−5.62, −2.38)	<0.001	−1.12
	6‐month follow‐up	21.2 (3.2)	28.2 (5.4)	−6.92 (−8.99, −4.85)	<0.001	−1.54
Cognitive confidence *F*(1.94, 144) = 3.64, *p* = 0.030	Baseline	17.6 (2.3)	18.4 (1.7)	−0.79 (−1.72, 0.14)	0.094	−0.39
	Post‐treatment	14.8 (2.7)	17.0 (1.2)	−2.13 (−3.10, −1.17)	<0.001	−1.01
	6‐month follow‐up	14.1 (1.8)	16.4 (2.0)	−2.37 (−3.23, −1.51)	<0.001	−1.25
Cognitive self‐consciousness *F*(2.00, 148) = 3.40, *p* = 0.036	Baseline	18.7 (2.0)	18.4 (1.7)	0.32 (−0.54, 1.17)	0.466	0.17
	Post‐treatment	16.1 (2.2)	16.6 (1.4)	−0.55 (−1.40, 0.30)	0.199	−0.28
	6‐month follow‐up	15.2 (2.4)	16.5 (1.8)	−1.32 (−2.28, −0.36)	0.008	−0.62
Negative beliefs about uncontrollability/danger *F*(1.90, 141) = 7.68, *p* < 0.001	Baseline	18.4 (3.5)	20.1 (1.0)	−1.68 (−2.86, −0.51)	0.006	−0.65
	Post‐treatment	15.3 (2.2)	18.5 (1.7)	−3.18 (−4.07, −2.30)	<0.001	−1.63
	6‐month follow‐up	14.0 (1.9)	18.2 (2.6)	−4.18 (−5.21, −3.15)	<0.001	−1.84
Need to control thoughts *F*(1.38, 102) = 8.37, *p* = 0.002	Baseline	16.4 (4.7)	17.6 (2.0)	−1.21 (−2.87, 0.45)	0.148	−0.33
	Post‐treatment	14.4 (1.7)	16.7 (1.9)	−2.29 (−3.10, −1.48)	<0.001	−1.28
	6‐month follow‐up	13.8 (1.3)	18.1 (1.7)	−4.29 (−4.96, −3.62)	<0.001	−2.81
Positive beliefs about worry *F*(1.94, 144) = 17.90, *p* < 0.001	Baseline	17.6 (2.3)	18.4 (1.7)	−0.79 (−1.72, 0.14)	0.094	−0.39
	Post‐treatment	14.4 (2.7)	16.5 (1.4)	−2.11 (−3.10, −1.11)	<0.001	−0.96
	6‐month follow‐up	14.1 (1.8)	18.3 (1.1)	−4.18 (−4.86, −3.51)	<0.001	−2.82

*Note:* Values are means and standard deviations from the analysis dataset and model‐estimated MCT minus TAU differences from REML mixed‐effects models. Negative differences and negative Hedges *g* values indicate lower scores in MCT relative to TAU. Higher scores indicate greater OCD severity or stronger endorsement of dysfunctional metacognitive beliefs.

Abbreviations: MCQ‐30, Metacognitions Questionnaire‐30; MCQ‐CC, cognitive confidence; MCQ‐CSC, cognitive self‐consciousness; MCQ‐NBW, negative beliefs about uncontrollability and danger; MCQ‐NCT, need to control thoughts; MCQ‐PBW, positive beliefs about worry; Y‐BOCS = Yale–Brown Obsessive‐Compulsive Scale.

#### 3.1.1. Harms and Adverse Events

No serious or treatment‐related adverse events were recorded in either condition during the acute treatment phase or the 6‐month follow‐up. There were no psychiatric hospitalizations, suicide attempts, treatment‐related clinical crises, medication emergencies, or treatment discontinuations attributable to adverse events. No important unintended harms were identified.

### 3.2. Treatment Effects on OCD Severity and Beliefs

Treatment effects are summarized in Table 2. The primary Y‐BOCS model showed a significant condition × time effect, indicating greater symptom reduction in MCT than in TAU across the three assessment waves. The groups showed little baseline separation on Y‐BOCS severity, whereas the between‐group differences favored MCT post‐treatment and were larger at the 6‐month follow‐up. Standardized between‐group effects were large at both endpoints. Categorical clinical outcomes are reported in Table 3. At post‐treatment, 7 of 38 participants (18.4%) in MCT and 0 of 38 (0.0%) in TAU met the prespecified response threshold of at least 35% reduction from baseline (risk difference = 18.4 percentage points, 95% CI [5.4, 33.4]). At follow‐up, response was observed in 13 of 38 participants (34.2%) in MCT and 6 of 38 (15.8%) in TAU (risk difference = 18.4 percentage points, 95% CI [−1.1, 36.4]); this estimate was imprecise, and the confidence interval included zero. No participant in either condition met the Y‐BOCS remission cutoff of ≤12 at either endpoint. The categorical findings therefore indicate clinically meaningful symptom improvement in a subset of participants but not full symptomatic remission.

**Table 3:** Y‐BOCS response and remission rates by condition at post‐treatment and follow‐up.

Outcome	Time point	MCT *n*/*N* (%)	TAU *n*/*N* (%)	Risk difference, pp (95% CI)	Risk ratio (95% CI)	Odds ratio (95% CI)
Response	Post‐treatment	7/38 (18.4%)	0/38 (0.0%)	18.4 (5.4, 33.4)	15.00 (0.89, 253.71)	18.33 (1.01, 333.60)
Response	6‐month follow‐up	13/38 (34.2%)	6/38 (15.8%)	18.4 (−1.1, 36.4)	2.17 (0.92, 5.10)	2.77 (0.92, 8.33)
Remission	Post‐treatment	0/38 (0.0%)	0/38 (0.0%)	0.0 (−9.2, 9.2)	NE	NE
Remission	6‐month follow‐up	0/38 (0.0%)	0/38 (0.0%)	0.0 (−9.2, 9.2)	NE	NE

*Note:* Response was defined as ≥35% reduction from baseline Y‐BOCS total score. Remission was defined as Y‐BOCS total score ≤12. Because CGI‐I/CGI‐S ratings were not collected, these outcomes represent the Y‐BOCS components of the consensus definitions. *N* = randomized participants per condition in the repeated‐measures intention‐to‐treat analysis dataset. Risk differences are MCT minus TAU and are reported in percentage points with Newcombe‐Wilson 95% confidence intervals. Risk ratios and odds ratios are reported with 95% confidence intervals; the Haldane‐Anscombe correction was applied when one condition had no events. NE = not estimable because no remission event occurred in either condition.

For the MCQ‐30 domains, the mixed‐effects models also showed significant condition × time effects, with the estimated differences generally favoring MCT at post‐treatment and follow‐up (Table 2). The most pronounced follow‐up differences were observed for negative beliefs about uncontrollability and danger, the need to control thoughts, and positive beliefs about worry. Numerical details for each domain and time point are presented in Table 2.

### 3.3. Mediation Analysis

We next conducted an exploratory three‐wave PLS‐SEM panel mediation analysis to test whether post‐treatment metacognitive beliefs accounted for the treatment effect on follow‐up OCD severity. Table 4 reports the primary mediation findings, and Table S1 reports the full set of secondary path coefficients and explained variance estimates.

**Table 4:** Primary longitudinal mediation findings.

Effect	*β*	SE	*t*	*p*
Direct effect: group → Y‐BOCS severity (T2)	−1.342	0.162	8.290	<0.001
Total indirect effect: group → MCQ domains (T1) → Y‐BOCS severity (T2)	−0.007	0.135	0.051	0.960
Specific indirect effect via cognitive confidence (T1)	0.082	0.099	0.831	0.406
Specific indirect effect via cognitive self‐consciousness (T1)	−0.146	0.103	1.429	0.153
Specific indirect effect via negative beliefs about uncontrollability/danger (T1)	−0.065	0.123	0.532	0.595
Specific indirect effect via need to control thoughts (T1)	0.004	0.059	0.060	0.952
Specific indirect effect via positive beliefs about worry (T1)	0.118	0.076	1.553	0.120
Autoregressive path: Y‐BOCS severity (T1) → Y‐BOCS severity (T2)	0.259	0.097	2.660	0.008

*Note: β* = standardized path coefficient from the original sample estimate. The total indirect effect tested whether the five post‐treatment MCQ‐30 domains jointly transmitted the treatment effect to follow‐up Y‐BOCS severity. Treatment condition was coded 1 = MCT and 0 = TAU; negative *β* values indicate lower scores in MCT relative to TAU. Because the PLS‐SEM model showed limited global fit (SRMR = 0.22; NFI = 0.58), these mediation coefficients should be interpreted as exploratory and tentative. Full secondary paths and explained variance estimates are provided in Table S1.

The model showed that treatment assignment predicted lower post‐treatment scores on all five MCQ‐30 domains and retained a direct association with lower follow‐up Y‐BOCS severity. However, the hypothesized indirect effect through the five post‐treatment MCQ‐30 domains was not statistically supported: the total indirect effect was near zero, and none of the domain‐specific indirect effects reached statistical significance (Table 4). This finding is therefore interpreted as a lack of evidence for the specified lagged indirect pathway in this exploratory model, not as definitive evidence that metacognitive processes cannot mediate symptom change.

The model explained a substantial variance in follow‐up Y‐BOCS severity (*R* ^2^ = 0.848). The approximate global fit indices indicated limited fit (SRMR = 0.22; NFI = 0.58); however, NFI reflects covariance‐based ML assumptions not well suited to this variance‐based model [38], and all 18 model indicators showed significant departures from normality (Cramér‐von Mises test, all *p* < 0.05). To evaluate predictive validity directly, we conducted PLSpredict and CVPAT (Table S2). *Q* ^2^ predict values were weak or slightly negative for the post‐treatment mediators (range: −0.046 to 0.133) but positive for all follow‐up constructs, including follow‐up Y‐BOCS severity (*Q* ^2^ predict = 0.287). The CVPAT showed no significant predictive advantage over the naïve IA benchmark at the post‐treatment level (all *p* > 0.40), but a significant advantage at follow‐up for four of the five MCQ‐30 domains (*p* ≤ 0.003) and for follow‐up Y‐BOCS severity specifically (*p* = 0.035), with a significant overall model comparison (*p* = 0.023). These results indicate that, despite a limited approximate global fit, the model shows formally tested, significant predictive validity for the follow‐up wave, the theoretical target of the mediation hypothesis, while showing no predictive advantage over a naïve benchmark at the immediate post‐treatment level. The mediation path estimates are nonetheless reported as exploratory and tentative given the sample size, model complexity, and imputed follow‐up data.

## 4. Discussion

This multisite, assessor‐blinded RCT examined whether MCT improves OCD outcomes beyond TAU and whether changes in dysfunctional metacognitive beliefs temporally account for later symptom changes. Across mixed‐effects models, MCT produced substantially greater reductions in clinician‐rated OCD severity than TAU post‐treatment and maintained this advantage at the 6‐month follow‐up. MCT also led to greater decreases than TAU on four of the five metacognitive belief domains at post‐treatment, with all five domains favoring MCT and between‐group differences widening by the 6‐month follow‐up. In contrast, the temporally ordered three‐wave panel mediation model, which controlled for baseline beliefs, prior OCD severity, and the autoregressive stability of symptoms, did not support a lagged indirect effect of treatment on follow‐up OCD severity through post‐treatment metacognitive beliefs. Instead, none of the post‐treatment metacognitive belief domains significantly predicted follow‐up OCD severity once baseline beliefs, prior symptom severity, and the autoregressive symptom path were taken into account. Taken together, the two analytic approaches converge in showing that MCT improves symptoms and reduces dysfunctional metacognitive beliefs, but they diverge in whether the belief domains assessed here uniquely explain subsequent symptom change over the post‐treatment‐to‐follow‐up interval.

A key contribution of this study is that it disentangles two complementary questions often conflated in psychotherapy research: (a) whether a treatment works (i.e., its overall effects on patient outcomes over time) and (b) whether it works through a specific time‐ordered mechanism. Mixed‐effects models are well suited to estimating average symptom trajectories and treatment × time effects under ITT principles, even when repeated‐measures data are missing [39]. In contrast, longitudinal mediation models impose a stricter test of causal mechanisms by requiring that change in a proposed mediator precede and predict later change in the outcome, while prior outcome levels and alternative pathways are controlled [23–25].

Similar outcome‐mechanism dissociations have been observed in time‐ordered OCD studies, in which proposed cognitive or behavioral mediators changed during treatment but did not predict later symptom change after autoregressive symptom effects were modeled [27, 28]. Given the complexity of the panel mediation model and the study sample size, the absence of unique lagged mediation should be interpreted cautiously, and a type‐II error cannot be ruled out.

To interpret these domain‐specific belief changes, it is useful to return to the S‐REF model. Within this framework, metacognitive beliefs form part of the knowledge base that configures executive control and activates the CAS [9, 11]. Reductions in MCQ‐30 domains may therefore indicate a weakening of beliefs that sustain threat monitoring, worry or rumination, and rigid thought‐control efforts. Such changes may facilitate disengagement from neutralizing and suppression, greater tolerance of uncertainty during triggers, and corrective learning [9, 10]. Overall, the belief changes observed in this trial are consistent with the central S‐REF proposition that modifying metacognition can alter the self‐regulatory style that maintains OCD.

Metacognitive theory proposes that intrusive thoughts become clinically problematic when they are interpreted through maladaptive beliefs about their significance, danger, and controllability and when these appraisals trigger rigid coping responses [9, 13]. The following paragraphs consider each belief domain in relation to OCD‐related processes and the observed temporal patterns.

First, negative beliefs about the uncontrollability and danger of thoughts reflect an appraisal that intrusive cognitive events are threatening and impossible to control. This appraisal may amplify anxiety, promote vigilant threat monitoring, and create an urgency to neutralize intrusions [9, 13]. In this study, this domain showed the largest between‐group difference at post‐treatment and remained markedly lower in the MCT group at follow‐up, suggesting that MCT weakened catastrophic metacognitive appraisals of thoughts. Second, beliefs about the need to control thoughts reflect the perceived necessity of suppressing intrusive thoughts. Attempts to control thoughts can paradoxically increase their salience and sustain covert neutralizing responses [9]. Longitudinal evidence indicates that need‐to‐control beliefs may have distinct associations with OCD outcomes over time, making this a theoretically relevant treatment target [22]. In the present study, MCT significantly reduced need‐to‐control beliefs post‐treatment, and these reductions were largely maintained.

Third, cognitive confidence refers to trust in memory and cognitive functioning. Low cognitive confidence is closely associated with compulsive checking and difficulty feeling certain that a ritual has been completed adequately [40]. Participants receiving MCT showed improved cognitive confidence, which may help reduce checking tendencies. Fourth, cognitive self‐consciousness reflects excessive attention to one’s thoughts and continuous monitoring of cognitive activity. This self‐focused style may maintain obsessional difficulties by increasing the salience of intrusive thoughts and sustaining internal threat monitoring [9, 11]. Between‐group differences in cognitive self‐consciousness were not significant at post‐treatment but favored MCT at the 6‐month follow‐up, suggesting a delayed reduction in unhelpful thought monitoring [10]. Finally, positive beliefs about worry reflect the view that worry or rumination is useful or protective. Such beliefs may encourage perseverative thinking in response to intrusions [9]. MCT reduced positive worry‐related beliefs relative to TAU, which may reduce the tendency to engage in prolonged rumination. A cross‐sectional study of 167 Pakistani adults with OCD identified cognitive confidence and negative beliefs about the uncontrollability and danger of worry as the most central metacognitive nodes, whereas only positive beliefs about worry were uniquely associated with concurrent OCD severity in the regression model [41], underscoring that network centrality and concurrent severity associations do not establish the temporal mediation tested here.

One interpretation is that these broad metacognitive belief domains are partly state‐dependent; when symptoms abate and rituals subside, individuals may report a reduced sense of danger/uncontrollability of thoughts, a reduced need to control their thoughts, and greater confidence in their memory and cognition. In other words, as OCD symptoms improve, general metacognitive beliefs may improve as a downstream effect.

Another possibility is that changes in beliefs and symptoms occurred largely in parallel during treatment, leaving little residual symptom change for the beliefs to explain over the longer follow‐up period. This interpretation is compatible with evidence that metacognitive process measures can change during both exposure‐based treatment and MCT [15, 21]. Such overlap may reflect common learning processes operating across different therapies rather than a single unidirectional mediation pathway specific to metacognitive belief change.

This finding does not exclude a contributory causal role for metacognitive beliefs; rather, it indicates that the measures and assessment schedule used here did not identify a single lagged mechanism of change.

## 5. Theoretical Implications

The pattern of findings has direct implications for the metacognitive model of OCD [10, 11]. MCT produced durable reductions in both symptom severity and dysfunctional metacognitive beliefs, consistent with the model’s claim that metacognitions are modifiable and treatment‐relevant. However, the absence of a lagged indirect effect through the broad MCB domains suggests that these trait‐like belief scores may operate as correlates or downstream markers of clinical state rather than as the proximal causal processes specified by the theory. The S‐REF model locates the maintenance of disorder in moment‐to‐moment processes perseverative processing, threat monitoring, and maladaptive control strategies rather than in global belief endorsement alone [11], and OCD‐specific formulations emphasize appraisals of intrusions, beliefs about rituals, and stop criteria for terminating compulsions [12, 40]. The null mediation finding is therefore compatible with metacognitive theory if causal change is carried by these more proximal, disorder‐specific processes at time scales finer than the three assessment waves used here; detecting such change requires denser, session‐level measurement and designs matched to the true temporal grain of mediation [24, 25]. In this sense, the present results refine rather than refute the theory by narrowing where in the metacognitive system the operative mechanism of individual MCT should be sought.

## 6. Practical Implications

At the same time, the findings have practical implications for clinical assessment and case formulation. Because post‐treatment belief scores did not predict subsequent symptom changes beyond what was explained by post‐treatment symptom levels, clinicians and services should be cautious about using broad metacognitive belief scores as stand‐alone indicators of recovery or proxies for long‐term outcomes. Monitoring symptom severity and functional behavioral changes (such as reductions in rituals, avoidance, and reassurance seeking), alongside process indicators that are more proximal to the MCT’s specific targets (e.g., the patient’s ability to respond flexibly to intrusive thoughts), may provide a more accurate index of treatment progress than the current approach.

## 7. Limitations and Future Directions

This study has several limitations. First, the comparison condition was TAU rather than an established first‐line intervention such as ERP; therefore, the findings demonstrate the superiority of MCT over routine clinical care in this sample but do not establish its comparative efficacy against ERP. Second, follow‐up attrition reduced the available sample and may have limited power to detect smaller long‐term and indirect effects. Although mixed‐effects models used all available observations and missing data for the mediation analysis were handled under a missing‐at‐random assumption, bias remains possible if this assumption is not met [39]. Third, the mediation analysis included only three assessment waves, five broad self‐report belief domains, and a relatively complex model for the available sample. Together with the limited global model fit, these features mean that the null indirect effects should be interpreted as exploratory rather than as definitive evidence against a metacognitive mechanism. Future studies should use larger samples, more frequent within‐treatment assessments, and OCD‐specific process measures that more closely reflect the proposed therapeutic mechanisms [12, 15, 25]. Fourth, the study did not evaluate implementation outcomes or collect CGI‐I and CGI‐S ratings; consequently, no conclusions can be drawn about scalability or cost effectiveness, and the reported response and remission classifications were based only on Y‐BOCS criteria.

## 8. Conclusion

Individual MCT produced greater reductions than documented TAU in clinician‐rated OCD severity and dysfunctional metacognitive beliefs in this Pakistani outpatient sample, and the between‐group differences were maintained at the 6‐month follow‐up. A greater proportion of participants in MCT met the Y‐BOCS response threshold at both endpoints, although no participant in either condition met the Y‐BOCS remission threshold. The panel model did not support post‐treatment MCQ‐30 domains as lagged mediators of later symptom change, so the proposed metacognitive mechanism remains unconfirmed. These findings support individual MCT as a promising specialist outpatient treatment when trained clinicians and supervision are available. They do not establish superiority to ERP, scalability, cost‐effectiveness, suitability for task shifting, or population‐level improvement in access to OCD care. Larger prospectively designed trials with more frequent and treatment‐proximal process measures are needed to clarify whether metacognitive change precedes, follows, or accompanies symptom improvement.

## Author Contributions

**Saeed Anwar:** conceptualization, methodology, investigation, project administration, formal analysis, data curation, writing – original draft. **Romana Younas:** investigation, data curation, writing – review and editing. **Wejdan Deebani:** writing – review and editing. **Jiang Yingjie:** supervision, methodology, funding acquisition, project administration, writing – review and editing.

## Funding

The authors gratefully acknowledge the financial support provided by the National Natural Science Foundation of China (Grant 32271095), and the National Science and Technology Innovation 2030 Major Project (Grant 2021ZD0200536). The authors also thank the School of Psychology, Northeast Normal University, and the Key Laboratory of Cognitive and Brain Sciences.

## Disclosure

All authors read and approved the final manuscript. The funders had no role in the study design, data collection, data analysis, data interpretation, writing of the manuscript, or the decision to submit the manuscript for publication.

## Conflicts of Interest

The authors declare no conflicts of interest.

## Supporting Information

Additional supporting information can be found online in the Supporting Information section.

## Supporting information


**Supporting Information** Tables S1 and S2 are provided in a separate editable file. Table S1: Presents the full secondary PLS‐SEM path coefficients and explained variance estimates; Table S2: Presents the PLSpredict and cross‐validated predictive ability test (CVPAT) results. The CONSORT 2010 Checklist is provided as a separate supplementary file documenting the reporting of this randomized controlled trial.

## Data Availability

Data and materials that support the findings of this study are available from the corresponding author upon request. Access to the data is subject to ethical approval and compliance with participant confidentiality agreements due to the sensitive nature of clinical trial information.
