# Supplementary material for: Assessing the Impact of Metacognitive Therapy on OCD Severity and Beliefs: A Three‐Wave Multisite RCT With Panel Mediation Analysis
Source: Depress Anxiety. 2026 Oct 15;2026:5060244. doi: 10.1155/da/5060244 (PMC13670996; doi:10.1155/da/5060244)
Supplement: Supplementary file 1 [file DA-2026-5060244-s001.zip › Supplementary Tables S1 S2.docx]

Supplementary materials

Assessing the Impact of Metacognitive Therapy on OCD Severity and Beliefs: A Three-Wave Multisite RCT with Panel Mediation Analysis

Manuscript ID 5060244

**Supplementary Table S1: Full secondary PLS-SEM path coefficients and explained variance estimates**

| **Path** | ***β*** | ***SE*** | ***t*** | ***p*** | **95% BC CI** |
| --- | --- | --- | --- | --- | --- |
| Group (MCT=1, TAU=0) → MCQ-CC (T1) | -0.783 | 0.224 | 3.489 | < .001 | [-1.220, -0.338] |
| Group (MCT=1, TAU=0) → MCQ-CC (T2) | -0.358 | 0.148 | 2.409 | .016 | [-0.692, -0.114] |
| Group (MCT=1, TAU=0) → MCQ-CSC (T1) | -0.824 | 0.223 | 3.702 | < .001 | [-1.275, -0.404] |
| Group (MCT=1, TAU=0) → MCQ-CSC (T2) | -0.420 | 0.161 | 2.605 | .009 | [-0.780, -0.156] |
| Group (MCT=1, TAU=0) → MCQ-NBW (T1) | -1.409 | 0.109 | 12.957 | < .001 | [-1.619, -1.187] |
| Group (MCT=1, TAU=0) → MCQ-NBW (T2) | -1.099 | 0.124 | 8.875 | < .001 | [-1.337, -0.855] |
| Group (MCT=1, TAU=0) → MCQ-NCT (T1) | -0.941 | 0.165 | 5.717 | < .001 | [-1.248, -0.601] |
| Group (MCT=1, TAU=0) → MCQ-NCT (T2) | -0.532 | 0.150 | 3.550 | < .001 | [-0.838, -0.258] |
| Group (MCT=1, TAU=0) → MCQ-PBW (T1) | -1.023 | 0.163 | 6.271 | < .001 | [-1.329, -0.697] |
| Group (MCT=1, TAU=0) → MCQ-PBW (T2) | -0.275 | 0.148 | 1.860 | .063 | [-0.610, -0.037] |
| Group (MCT=1, TAU=0) → Y-BOCS (T2) | -1.351 | 0.178 | 7.580 | < .001 | [-1.671, -0.965] |
| MCQ-CC (T0) → MCQ-CC (T1) | 0.155 | 0.118 | 1.312 | .190 | [-0.090, 0.371] |
| MCQ-CC (T0) → MCQ-CC (T2) | 0.071 | 0.058 | 1.215 | .224 | [-0.040, 0.192] |
| MCQ-CC (T0) → Y-BOCS (T2) | -0.016 | 0.025 | 0.659 | .510 | [-0.073, 0.031] |
| MCQ-CC (T1) → MCQ-CC (T2) | 0.457 | 0.078 | 5.835 | < .001 | [0.295, 0.606] |
| MCQ-CC (T1) → Y-BOCS (T2) | -0.105 | 0.117 | 0.893 | .372 | [-0.344, 0.122] |
| MCQ-CSC (T0) → MCQ-CSC (T1) | 0.234 | 0.106 | 2.216 | .027 | [0.023, 0.440] |
| MCQ-CSC (T0) → MCQ-CSC (T2) | 0.119 | 0.054 | 2.209 | .027 | [0.012, 0.225] |
| MCQ-CSC (T0) → Y-BOCS (T2) | 0.042 | 0.034 | 1.209 | .227 | [-0.011, 0.121] |
| MCQ-CSC (T1) → MCQ-CSC (T2) | 0.510 | 0.078 | 6.526 | < .001 | [0.349, 0.656] |
| MCQ-CSC (T1) → Y-BOCS (T2) | 0.178 | 0.113 | 1.575 | .115 | [-0.040, 0.409] |
| MCQ-NBW (T0) → MCQ-NBW (T1) | 0.212 | 0.068 | 3.106 | .002 | [0.072, 0.339] |
| MCQ-NBW (T0) → MCQ-NBW (T2) | 0.165 | 0.055 | 2.983 | .003 | [0.055, 0.272] |
| MCQ-NBW (T0) → Y-BOCS (T2) | 0.010 | 0.019 | 0.524 | .600 | [-0.028, 0.048] |
| MCQ-NBW (T1) → MCQ-NBW (T2) | 0.780 | 0.050 | 15.652 | < .001 | [0.671, 0.865] |
| MCQ-NBW (T1) → Y-BOCS (T2) | 0.046 | 0.086 | 0.542 | .588 | [-0.123, 0.213] |
| MCQ-NCT (T0) → MCQ-NCT (T1) | 0.195 | 0.094 | 2.071 | .038 | [0.007, 0.378] |
| MCQ-NCT (T0) → MCQ-NCT (T2) | 0.110 | 0.052 | 2.116 | .034 | [0.003, 0.209] |
| MCQ-NCT (T0) → Y-BOCS (T2) | -0.001 | 0.013 | 0.056 | .955 | [-0.025, 0.030] |
| MCQ-NCT (T1) → MCQ-NCT (T2) | 0.565 | 0.078 | 7.255 | < .001 | [0.396, 0.698] |
| MCQ-NCT (T1) → Y-BOCS (T2) | -0.004 | 0.060 | 0.063 | .950 | [-0.122, 0.113] |
| MCQ-PBW (T0) → MCQ-PBW (T1) | 0.226 | 0.100 | 2.250 | .024 | [0.030, 0.427] |
| MCQ-PBW (T0) → MCQ-PBW (T2) | 0.061 | 0.034 | 1.764 | .078 | [0.002, 0.135] |
| MCQ-PBW (T0) → Y-BOCS (T2) | -0.026 | 0.019 | 1.340 | .180 | [-0.072, 0.002] |
| MCQ-PBW (T1) → MCQ-PBW (T2) | 0.269 | 0.112 | 2.404 | .016 | [0.048, 0.485] |
| MCQ-PBW (T1) → Y-BOCS (T2) | -0.115 | 0.067 | 1.727 | .084 | [-0.250, 0.009] |
| Y-BOCS (T0) → Y-BOCS (T1) | 0.165 | 0.118 | 1.396 | .163 | [-0.078, 0.389] |
| Y-BOCS (T0) → Y-BOCS (T2) | 0.043 | 0.037 | 1.158 | .247 | [-0.020, 0.125] |
| Y-BOCS (T1) → Y-BOCS (T2) | 0.259 | 0.097 | 2.660 | .008 | [0.086, 0.470] |
| Explained variance (*R²*) |  |  |  |  |  |
| MCQ-CC (T1) | 0.201 |  |  | .039 | *R²* |
| MCQ-CC (T2) | 0.209 |  |  | .003 | *R²* |
| MCQ-CSC (T1) | 0.206 |  |  | .037 | *R²* |
| MCQ-CSC (T2) | 0.260 |  |  | .001 | *R²* |
| MCQ-NBW (T1) | 0.636 |  |  | < .001 | *R²* |
| MCQ-NBW (T2) | 0.608 |  |  | < .001 | *R²* |
| MCQ-NCT (T1) | 0.288 |  |  | .001 | *R²* |
| MCQ-NCT (T2) | 0.319 |  |  | < .001 | *R²* |
| MCQ-PBW (T1) | 0.257 |  |  | .001 | *R²* |
| MCQ-PBW (T2) | 0.072 |  |  | .241 | *R²* |
| Y-BOCS (T1) | 0.027 |  |  | .518 | *R²* |
| Y-BOCS (T2) | 0.848 |  |  | < .001 | *R²* |

Note. Supplementary Table S1 provides the secondary longitudinal paths and explained variance estimates from the exploratory PLS-SEM panel model. BC CI = bias-corrected bootstrap confidence interval. T0 = baseline; T1 = post-treatment; T2 = 6-month follow-up. MCQ-CC = cognitive confidence; MCQ-CSC = cognitive self-consciousness; MCQ-NBW = negative beliefs about uncontrollability and danger; MCQ-NCT = need to control thoughts; MCQ-PBW = positive beliefs about worry. These secondary paths are reported for transparency and should not be interpreted as confirmatory evidence of mechanism because of model complexity, follow-up attrition and imputation, and limited global fit.

**Supplementary Table S2: PLSpredict and CVPAT results**

| **Construct** | ***Q²predict*** | **PLS-SEM RMSE** | **LM RMSE** | **CVPAT *p* (vs. IA)** |
| --- | --- | --- | --- | --- |
| MCQ-CC (T1) | -0.001 | 2.409 | 2.354 | .996 |
| MCQ-CC (T2) | 0.241 | 2.254 | 1.628 | < .001 |
| MCQ-CSC (T1) | -0.024 | 2.223 | 2.023 | .886 |
| MCQ-CSC (T2) | 0.203 | 2.380 | 1.750 | .001 |
| MCQ-NBW (T1) | 0.133 | 2.378 | 1.684 | .405 |
| MCQ-NBW (T2) | 0.232 | 2.935 | 2.358 | .051 |
| MCQ-NCT (T1) | 0.031 | 1.984 | 1.785 | .831 |
| MCQ-NCT (T2) | 0.285 | 2.214 | 1.565 | < .001 |
| MCQ-PBW (T1) | -0.046 | 1.674 | 1.504 | .756 |
| MCQ-PBW (T2) | 0.124 | 2.479 | 1.921 | .003 |
| Y-BOCS (T1) | -0.006 | 6.396 | 3.851 | .888 |
| Y-BOCS (T2) | 0.287 | 6.358 | 3.781 | .035 |
| **Overall model** | **—** | **—** | **—** | **.023** |

Note. Q²predict, RMSE, and LM benchmark values are from PLSpredict. CVPAT p values test the model’s average prediction loss against an indicator-average (IA) benchmark. T1 = post-treatment; T2 = 6-month follow-up.
