# Supplementary material for: Assessing the Impact of Metacognitive Therapy on OCD Severity and Beliefs: A Three‐Wave Multisite RCT With Panel Mediation Analysis
Source: Depress Anxiety. 2026 Oct 15;2026:5060244. doi: 10.1155/da/5060244 (PMC13670996; doi:10.1155/da/5060244)
Supplement: Supplementary file 1 [file DA-2026-5060244-s001.zip › CONSORT 2010 Checklist Final.docx]

**CONSORT 2010 Checklist**
Assessing the Impact of Metacognitive Therapy on OCD Severity and Beliefs: A Three-Wave Multisite RCT with Panel Mediation Analysis

Page references correspond to the final 33-page manuscript. NR = not reported; N/A = not applicable. References to Tables S1 and S2 refer to the separate supplementary file.

| **Section/Topic** | **Item No.** | **Checklist item** | **Reported on page No.** |
| --- | --- | --- | --- |
| **Title and abstract** | | | |
| Title | 1a | Identification as a randomised trial in the title | 1–2 |
| Abstract | 1b | Structured summary of trial design, methods, results, and conclusions (for specific guidance, see CONSORT for abstracts) | 2 (summary provided without structured subheadings) |
| **Introduction** | | | |
| Background and objectives | 2a | Scientific background and explanation of rationale | 3–6 |
| Background and objectives | 2b | Specific objectives or hypotheses | 6–7 |
| **Methods** | | | |
| Trial design | 3a | Description of trial design (such as parallel, factorial), including allocation ratio | 7, 10 |
| Trial design | 3b | Important changes to methods after trial commencement (such as eligibility criteria), with reasons | NR (changes to methods not explicitly stated in the manuscript) |
| Participants | 4a | Eligibility criteria for participants | 8 |
| Participants | 4b | Settings and locations where the data were collected | 7 |
| Interventions | 5 | The interventions for each group with sufficient details to allow replication, including how and when they were actually administered | 9–12 |
| Outcomes | 6a | Completely defined pre-specified primary and secondary outcome measures, including how and when they were assessed | 7–9, 14 |
| Outcomes | 6b | Any changes to trial outcomes after the trial commenced, with reasons | No changes to trial outcomes; not explicitly stated in the manuscript |
| Sample size | 7a | How the sample size was determined | 15 |
| Sample size | 7b | When applicable, an explanation of any interim analyses and stopping guidelines | N/A |
| Randomisation: Sequence generation | 8a | Method used to generate the random allocation sequence | 10 |
| Randomisation: Sequence generation | 8b | Type of randomization; details of any restriction (such as blocking and block size) | 10 (block randomisation; block size NR) |
| Randomisation: Allocation concealment mechanism | 9 | Mechanism used to implement the random allocation sequence (such as sequentially numbered containers), describing any steps taken to conceal the sequence until interventions were assigned | 10 |
| Randomisation: Implementation | 10 | Who generated the random allocation sequence, who enrolled participants, and who assigned participants to interventions | 7, 10 (sequence generator and recruitment described; enrolment and assignment personnel not fully specified) |
| Blinding | 11a | If done, who was blinded after assignment to interventions (for example, participants, care providers, those assessing outcomes) and how | 9–10 |
| Blinding | 11b | If relevant, a description of the similarity of interventions | 10–12 (both arms: 12 weekly individual sessions of 45–60 minutes) |
| Statistical methods | 12a | Statistical methods used to compare groups for primary and secondary outcomes | 13–14 |
| Statistical methods | 12b | Methods for additional analyses, such as subgroup analyses and adjusted analyses | 14–15 |
| **Results** | | | |
| Participant flow | 13a | For each group, the number of participants who were randomly assigned, received intended treatment, and were analyzed for the primary outcome | 8, 15–16; Figure 1 (29) |
| Participant flow | 13b | For each group, losses and exclusions after randomization, together with reasons | 15; Figure 1 (29) |
| Recruitment | 14a | Dates defining the periods of recruitment and follow-up | 9, 12 (January 2024 study start and 6-month follow-up reported; full recruitment and follow-up calendar dates NR) |
| Recruitment | 14b | Why the trial ended or was stopped | N/A (completed trial; 12) |
| Baseline data | 15 | A table showing baseline demographic and clinical characteristics for each group | 15; Table 1 (30–31); Table 2 (31–32) |
| Numbers analysed | 16 | For each group, the number of participants (denominator) included in each analysis and whether the analysis was by original assigned groups | 13–16; Figure 1 (29); Table 3 (33) |
| Outcomes and estimation | 17a | For each primary and secondary outcome, results for each group, and the estimated effect size and its precision (such as 95% confidence interval) | 16–17; Tables 2–4 (31–33); Supplementary Table S1 |
| Outcomes and estimation | 17b | For binary outcomes, presentation of both absolute and relative effect sizes is recommended | 16; Table 3 (33) |
| Ancillary analyses | 18 | Results of any other analyses performed, including subgroup analyses and adjusted analyses, distinguishing pre-specified from exploratory | 17; Table 4 (33); Supplementary Tables S1–S2 (exploratory panel and predictive analyses) |
| Harms | 19 | All important harms or unintended effects in each group (for specific guidance, see CONSORT for harms) | 16 (no serious or treatment-related adverse events); monitoring: 9–10 |
| **Discussion** | | | |
| Limitations | 20 | Trial limitations, addressing sources of potential bias, imprecision, and, if relevant, multiplicity of analyses | 18, 22 |
| Generalisability | 21 | Generalisability (external validity, applicability) of the trial findings | 18, 21–22 |
| Interpretation | 22 | Interpretation consistent with results, balancing benefits and harms, and considering other relevant evidence | 18–22; harms: 16 |
| **Other information** | | | |
| Registration | 23 | Registration number and name of trial registry | 12 (ClinicalTrials.gov: NCT07052812; retrospective registration) |
| Protocol | 24 | Where the full trial protocol can be accessed, if available | 12–13 (available from the corresponding author upon reasonable request) |
| Funding | 25 | Sources of funding and other support (such as supply of drugs), role of funders | 23 |
